# DIY liquid handling robots for integrated STEM education and life science research

**DOI:** 10.1371/journal.pone.0275688

**Published:** 2022-11-09

**Authors:** Ethan Li, Amy T. Lam, Tamar Fuhrmann, Len Erikson, Mike Wirth, Mark L. Miller, Paulo Blikstein, Ingmar H. Riedel-Kruse

**Affiliations:** 1 Department of Bioengineering, Stanford University, Stanford, California, United States of America; 2 The Miller Institute for Learning with Technology, San Carlos, California, United States of America; 3 Teachers College, Columbia University, New York, New York, United States of America; 4 Department of Molecular and Cellular Biology, Departments of Applied Mathematics, Biomedical Engineering, and Physics, University of Arizona, Tucson, Arizona, United States of America; Istituto Italiano di Tecnologia, ITALY

## Abstract

Automation has played a key role in improving the safety, accuracy, and efficiency of manufacturing and industrial processes and has the potential to greatly increase throughput in the life sciences. However, the lack of accessible entry-point automation hardware in life science research and STEM education hinders its widespread adoption and development for life science applications. Here we investigate the design of a low-cost (~$150) open-source DIY Arduino-controlled liquid handling robot (LHR) featuring plastic laser-cut parts. The robot moves in three axes with 0.5 mm accuracy and reliably dispenses liquid down to 20 μL. The open source, modular design allows for flexibility and easy modification. A block-based programming interface (Snap4Arduino) further extends the accessibility of this robot, encouraging adaptation and use by educators, hobbyists and beginner programmers. This robot was co-designed with teachers, and we detail the teachers’ feedback in the context of a qualitative study. We conclude that affordable and accessible LHRs similar to this one could provide a useful educational tool to be deployed in classrooms, and LHR-based curricula may encourage interest in STEM and effectively introduce automation technology to life science enthusiasts.

## Introduction

Automation of life science experiments and protocols is important for increasing efficiency and accuracy for various applications, ranging from the manufacturing of various bio-based products, such as pharmaceuticals and biofuels, to biochemical research assays [1–3]. Such products form the basis of the emerging bioeconomy, which is expected to be the next great frontier in economic development [4,5]. Because of the rising importance of automation and robotics in biological assays [1], increasing the general accessibility of and interest in these tools within the context of the life sciences could increase engagement and accelerate further development. Increased accessibility can be achieved through developing low-cost toolkits and robots, along with user-friendly interfaces, and could play an important role in educating the future bioeconomy workforce as well as enable citizen science, cloud laboratories, and rapid technological innovations [3–7].

Due to the nature of biological samples and experiments, automation will necessarily involve liquid handling robots (LHRs), which must be capable of handling minute quantities of liquids accurately and without risk of contamination (i.e., pipetting) [2,8]. Furthermore, the frequency and repetitiveness of pipetting tasks make them an ideal target for automation. However, currently, commercially deployed liquid handling robots are expensive (on the order of thousands to tens of thousands of U.S. dollars) and are typically designed to conduct specific standard protocols [9–11]. These shortcomings limit the usability of such robots for more exploratory, non-standard work as well as the accessibility of these robots for researchers and the general public.

Developing low-cost, easy-to-use, open-source, and safe LHRs could greatly increase accessibility to automation technology in the life sciences [2,3,8,12]. Lowering the cost of LHRs would allow them to be more widely employed in laboratory settings, which would in turn enable researchers to dedicate more time to higher-level aspects of lab work and training. Low-cost robots could also be employed in more informal and non-professional settings, such as in classrooms for STEM education or even at home, enabling greater public engagement with biology. In particular, availability of low-cost LHRs would be valuable for fostering interdisciplinary STEM education because LHRs bridge the areas of biology, computer science and programming, and device engineering and robotics [3]. Such interdisciplinary education is recommended by the Next Generation Science Standards (NGSS) as well as other national initiatives for STEM education due to the increasingly cross-disciplinary nature of research and innovative technologies [13]. Furthermore, “do-it-yourself” (DIY) low-cost tools can serve as an entry point to life science research for hobbyists and other bio-enthusiasts and increase the design space for artists, educators, and researchers [5,14].

A number of DIY LHRs have been developed previously. OpenLH [8] featured custom 3D printed parts integrated with commercially available robotic parts to achieve a minimum droplet size of 0.2 μL with a standard deviation of 0.15 μL at a price point of about $1,000. EvoBot [15,16] also featured 3D printed parts combined with laser-cut parts placed within an aluminum frame. The parts were designed to be modular to enable easy modifications and extensions to be made to the robot. EvoBot dispensed liquid down to ~10 μL at a price point of <$500 for the hardware. However, the assembly of the EvoBot was noted to be difficult, requiring knowledge of robotics. The Lego Liquid Handler system [3] used a different approach to enable DIY construction: built almost entirely with the standard Lego EV3 Education kit, the burden of producing the basic building blocks and electronics is eliminated for the user. The user gains experience in assembling the robot structure by snapping the Lego pieces in place and in programming through the EV3 controller and software. However, because it uses a preexisting kit, the user cannot completely customize the setup and software. This Lego Liquid Handler had a price point of <$450, making it the cheapest of these DIY sets and had a minimum droplet size of 2.5 μL, and could reliably dispense volumes as little as 0.15 μL with a specialized syringe. All of these DIY robots were designed to be able to handle standard laboratory parts such as plastic cuvettes, 96-well plates, a syringe, and pipette tips.

In comparison, the Opentrons system, which costs approximately $5,000 for the basic setup, is the cheapest step up for liquid handling systems [9–11]. Opentrons is open-source and is simple enough for users to assemble themselves for use in a laboratory setting to conduct standard protocols with standard labware [17]. It is far more affordable than most industry standard liquid handlers, which run in the tens of thousands of dollars. However, its price point is still relatively high, which discourages its use for science education or prototyping.

In addition to decreasing costs, developing user-friendly interfaces for robotic operation and programming can also increase accessibility. One avenue is to modify user interfaces that people are already familiar with to work with liquid handling machines, as was done in [12], where a desktop printer and scanner were modified to handle biological fluids. Another option is to use open-source software, as Opentrons does [17], which makes it easy to find and share protocols and tailor them as needed. However, software modification still often requires some programming experience. A third option is to use block-based programming interfaces, where the programs are graphically represented through modular icons and can be easily arranged and rearranged by the user. This was done in the OpenLH [8] system, which had both a traditional programming interface (a Python API), as well as a block-based programming interface. Block-based programming interfaces are often used as educational tools for novice programmers, and can also make it easier for users to extend the interface to promote creative exploration [8].

**Fig 1:**
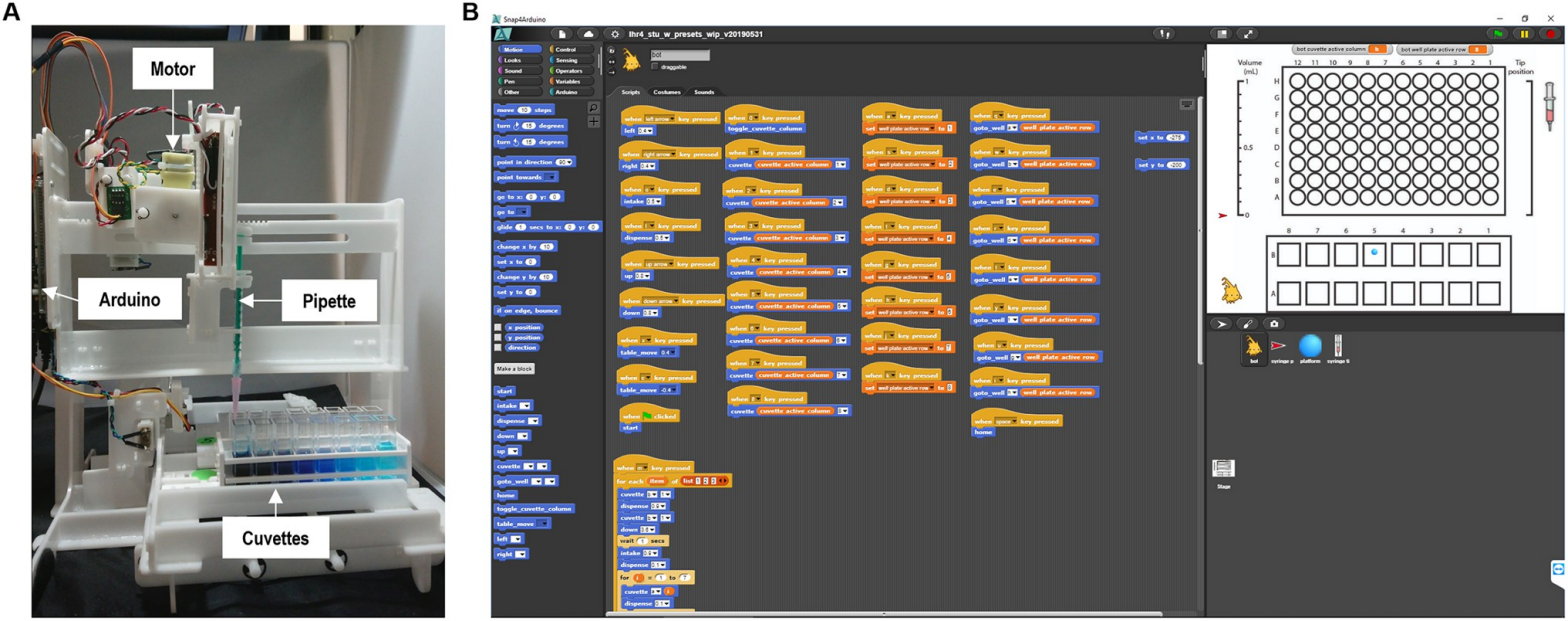
An open-source low-cost liquid handling robot (LHR) for STEM education and life-science experimentation featuring a block-based programming language. **A)** The main frame of the robot is made of laser-cut plastic parts, while all consumables (i.e. syringes, pipette tips, cuvettes, and well-plates) are standard life-science plastic ware that can be purchased online. **B)** The LHR interface was programmed in Snap4Arduino. (See also Supplementary Movies and reference [21]).

Here we present an open-source robot which can be created from the “ground up” at low-cost and employs an open block-based programming interface (Fig 1, Supplementary Movies). Our rationale for creating such a robot is to increase the transparency of the manufacturing process of the robot, helping users build a better understanding of the system; add more customizability so that users can adapt the robot to their individual use-case; and have this robot serve as an introduction to multiple fields such as programming, manufacturing, life sciences, and chemistry. Our goals were (1) to design an LHR that can easily be assembled with snap-together laser-cut parts, which are easily modified from open-source blueprints (i.e., CAD files) and is compatible with standard lab plasticware (e.g., 96-well plates); (2) to use inexpensive open-source DIY electronics such as Arduino boards [18,19]; (3) to enable distributed manufacturing (i.e., each individual user can manufacture the parts locally for themselves) [20], (4) to integrate this robot fully with a block-based programming language to enable non-specialist users to operate and automate protocols; (5) to characterize the performance parameters of this robot, and (6) to take into account the perspective of potential future users (i.e., teachers) through a joint and iterative co-design process. We first describe the design of the full robot and the programming interface. Then, we characterize the robot’s performance and assess its functionality with automated serial dilution and cell seeding protocols. We conclude with a discussion on the key accomplishments and contributions for this work with an outlook for future improvements and extensions. Here, we focus on aspects of LHR performance and design; an educational study to assess how school children can use this particular LHR has been published elsewhere [21].

## Results

### Hardware design

Although our first prototype robot operated along a single axis (S4 Movie), our final implementation of the robot had three axes of motion for *x-y-z* positioning and a fourth linear actuator for syringe plunger actuation (Fig 1A, S1 and S2 Movies). This final design is described here (and in the Supplementary Materials) in detail.

**Fig 2:**
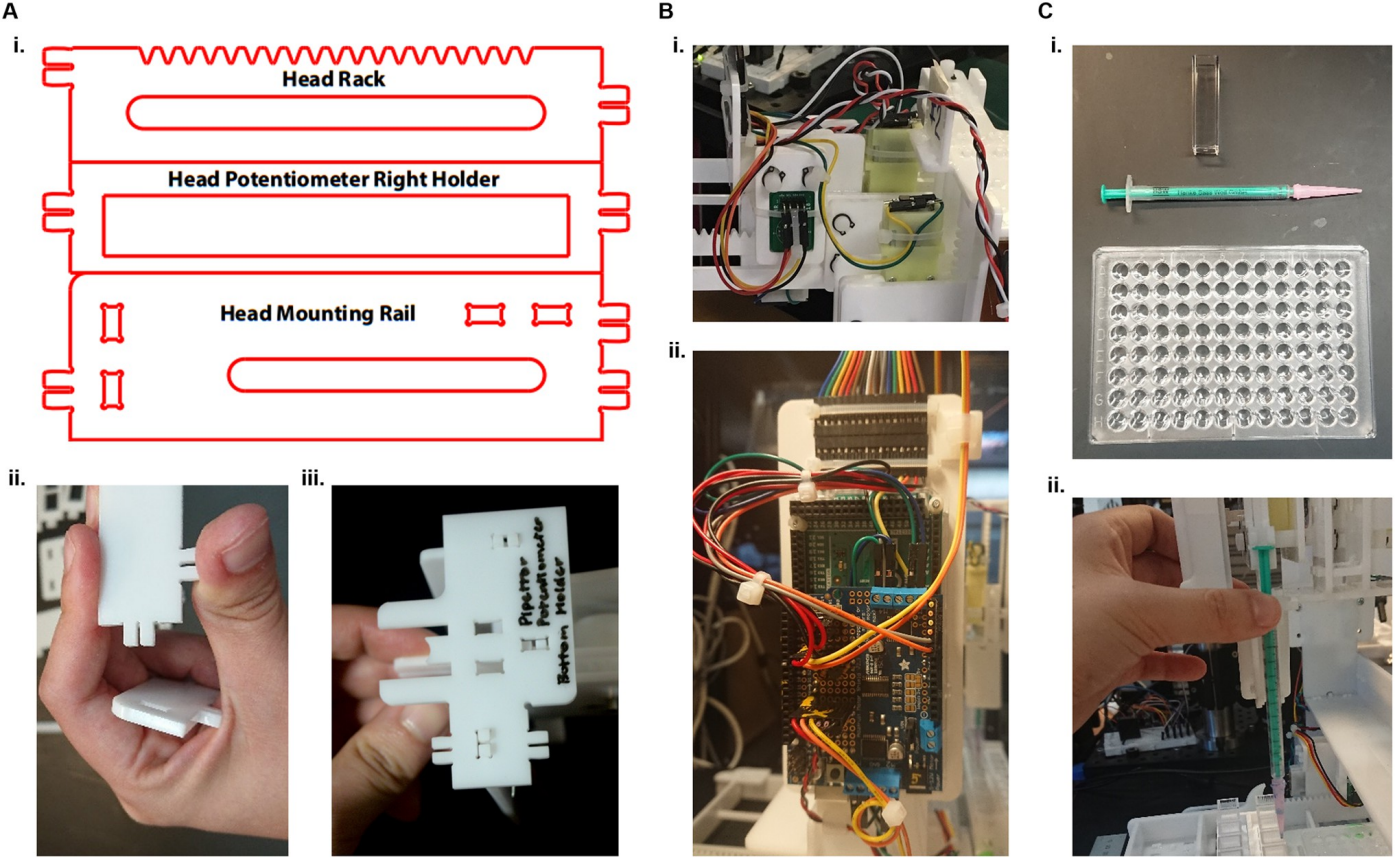
Close up of various parts of the robot. **A)** The laser-cut parts. i. Examples of the laser cutting patterns for parts found on the sheets (see Supplemental Information for all laser-cut sheets). ii. The laser-cut parts have tabs which can be snapped into rectangular holes. iii. The tabs can be seen partially inserted into holes. The relative sizes of the tabs and holes need to be adjusted for each laser cutter to be able to fit well. **B)** i. The motors are held in place with the laser-cut parts and wired to the Arduino board. ii. The Arduino board is mounted onto the left side of the robot and controls the motors. **C)** i. The consumables: Standard 1 cm cuvette, a syringe with a disposable tip, and a 96-well plate. ii. The standard plasticware can be slotted into place.

Our positioning mechanism consists of an *x-z* gantry to position the syringe in two axes of motion and a *y*-axis motion stage to position samples in the remaining axis of motion (Fig 1A). We wished to design the robot to enable repeated disassembly and reassembly, with all parts joined by either snap-fit tab-hole joints or press-fit holes (Fig 2A). Our design objectives were to prioritize structural rigidity and stability, followed by modularity, and low parts count. The robot’s mechanical structure consists almost entirely of laser-cut acrylic pieces and hand-cut Delrin rods, with the exception of a few small 3D printed ABS plastic parts; all raw materials are available from McMaster-Carr and thus accessible to hobbyists and non-specialists (Supplementary Materials).

For position control of the robot’s four linear actuators, we use low-cost brushed DC motors with rack-and-pinion mechanisms and position sensors (Fig 2B(i)). For position sensing, we use linear potentiometers when the motion range requirements were sufficiently small, and we use magnetic angle sensors when we required large motion ranges (Supplementary Materials). Position control of the robot’s linear actuators is implemented on an Arduino microcontroller board (Fig 2B(ii)), which is connected to the motors and position sensors via an Adafruit motor shield. With the exception of the magnetic angle sensors, all components are available from hobbyist-oriented vendors (Adafruit, Sparkfun, Pololu, and Jameco). The magnetic angle sensors require a few parts from a business-oriented vendor (Digikey), integrated into two custom printed circuit boards.

**Fig 3:**
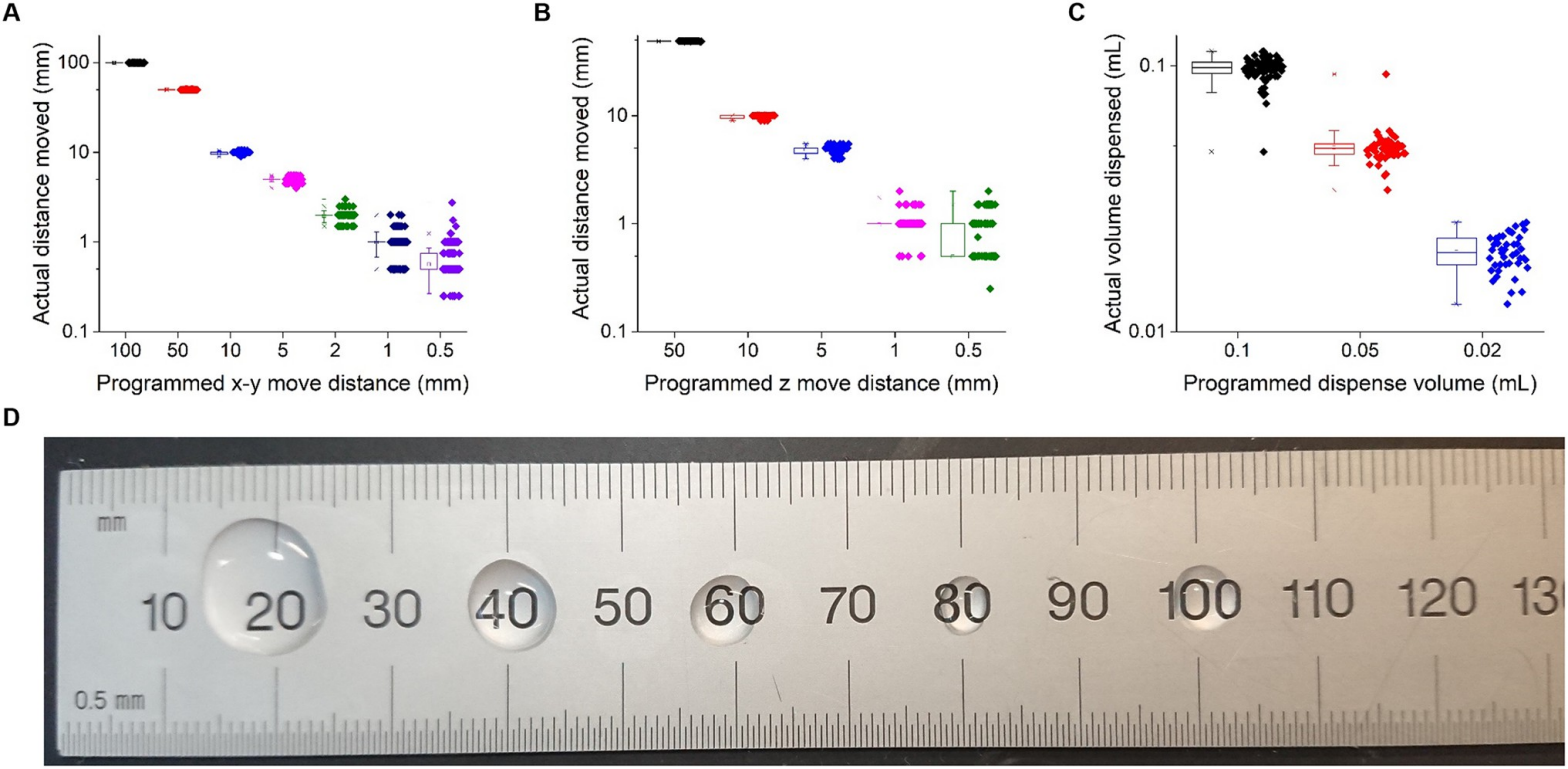
The LHR is accurate down to 5 mm of motion with a standard deviation of 0.5 mm and can dispense as little as 50 μL with a standard deviation of 10 μL. **A)** Motor accuracy for x-y motion. The x-axis is the programmed distance for the motor to move, and the y-axis is the actual distance moved by the motor. N = 20, 46, 62, 80, 103, 204, and 359 for the programmed move distances of 100 mm, 50 mm, 10 mm, 5 mm, 2 mm, 1 mm, and 0.5 mm, respectively. **B)** Motor accuracy for z motion. The x-axis is the programmed distance for the motor to move, and the y-axis is the actual distance moved by the motor. N = 20, 20, 40, 100, and 194 for programmed move distances of 50 mm, 10 mm, 5 mm, 1 mm, and 0.5 mm, respectively. **C)** Motor accuracy for liquid dispensing volume. The x-axis is the programmed volume for the robot to dispense, and the y-axis is the actual volume of liquid dispensed. N = 71, 50, and 43 for programmed dispense volumes of 0.1 mL, 0.05 mL, and 0.02 mL, respectively. **D)** Image of droplet sizes over multiple dispense commands of various droplet volumes at various locations along a line. At every other cm mark, the robot dispenses (from left to right) 0.1 mL, 50 μL, 25 μL, 10 μL, and 5 μL. Note that the 5 μL droplet appears to be the same size or perhaps even larger than the 10 μL droplet, indicating that the robot is not accurate down to this volume. For A)-C), the plots contain the box plot summarizing the data to the left of each set of individual data points. For the box plots, the whiskers denote the range between the outlier boundaries (± 1.5 times the interquartile range), the box boundaries denote the 25^th^ and 75^th^ percentile, and the central square data point in the box plot denotes the mean.

In total, the robot has 4 degrees of motion (Fig 1A, Supplementary Movies). The *y*-axis motor shifts the entire stage towards or away from the user and has an arbitrarily extensible range of motion. This allows for the modular design of the stage, which enables users to add various platforms for their experimental needs. The *x*-axis motor moves the pipette holder (Fig 2C) left or right, while the *z*-axis motor raises and lowers the pipette away from or towards the sample, respectively. This allows the pipette tip to be positioned into and out of well plates, cuvettes, petri dishes, and other types of containers used in experimental protocols. Finally, the pipettor axis motor raises and lowers the syringe pump thus regulating the intake and dispensing of the liquid (S1 Movie). The *x*-axis motor has a 14 cm range of motion while both the *z*-axis motor and the pipettor axis motor have a 5.2 cm range of motion. Thus, the syringe can intake up to approximately 0.9 mL of liquid. The *x*-*y* stage as well as the pipette tip can travel 5 mm with greater than 10% accuracy (Fig 3A and 3B). The robot can also dispense liquid down to 50 μL with greater than 20% accuracy (Fig 3C and 3D).

The stage has several modules which were designed to fit standard wet lab plasticware (Figs 1A and 2C, Supplementary Materials). For example, one module holds 16 standard cuvettes while another holds a 96-well plate. The plasticware fits with <1 mm tolerance to ensure minimal shifting during stage movement, which would affect the calibration. The travel distance of the *z*-axis motor is large enough to insert and completely remove the pipette tip from the standard plasticware so that liquid can be taken in and dispensed directly into the container or well. This platform can handle glass or plasticware with the height of the container up to 5 cm.

### Hardware reproducibility

We wished to determine if an easily assembled, reproducible, and modifiable DIY robot could be built. To that end, once the design was finalized, we built five replicas of the prototype robot. The replication process was straightforward, with a typical assembly and building time of about 1 day for each additional robot for research personnel already familiar with the design. From this process, we learned that one of the key challenges to replicating the robot was that the motor encoders needed to be tuned for each individual robot. The five LHRs resulting from this process were used for educational user studies, the first of which we published previously [21].

To assess ease of future replication and determine how difficult it would be for a non-specialist to assemble the LHR, we gave the parts list and design plans to another researcher who had not been involved in the original design. This researcher ordered parts and recreated the robot over the course of about 3 working days (i.e., 24 hours). While most of the assembly proceeded as expected the researcher encountered two challenges: (1) Different vendors have slightly different plastic sheet thicknesses, and laser cutters can have variable cutting parameters (e.g., laser kerf), which can both lead to sub-optimal fittings. Thus, parameters such as hole and tab sizes should be tested and adjusted on a subset of parts before cutting the full set. Even with the additional tuning, laser cutting still results in some variation in tab/hole sizes, and additional gluing was necessary to maintain the structure of the robot. (2) The motor encoders need to be tuned for each robot which takes extra time and skill. We originally designed these encoders ourselves in the DIY spirit of building from the ground up and using inexpensive parts, but this came at the cost of having increased development time and greater challenges of reproduction. Future iterations of the LHR design should therefore instead use motors that already have built-in rotary encoders. Overall, we conclude that the reproduction of our robot design requires some skill and DIY experience. This is in contrast–unsurprisingly–to the previously published Lego liquid handling robots that could be assembled by advanced elementary school students [3].

### Control software

The robot control software (Fig 1B) has two independent modules, one running on the Arduino and one on a control computer. The Arduino’s software implements a position feedback control loop (PID controller) on each axis and exposes a serial communication interface to the control computer. The control computer software handles user interaction and sends higher-level movement commands to the Arduino.

**Fig 4:**
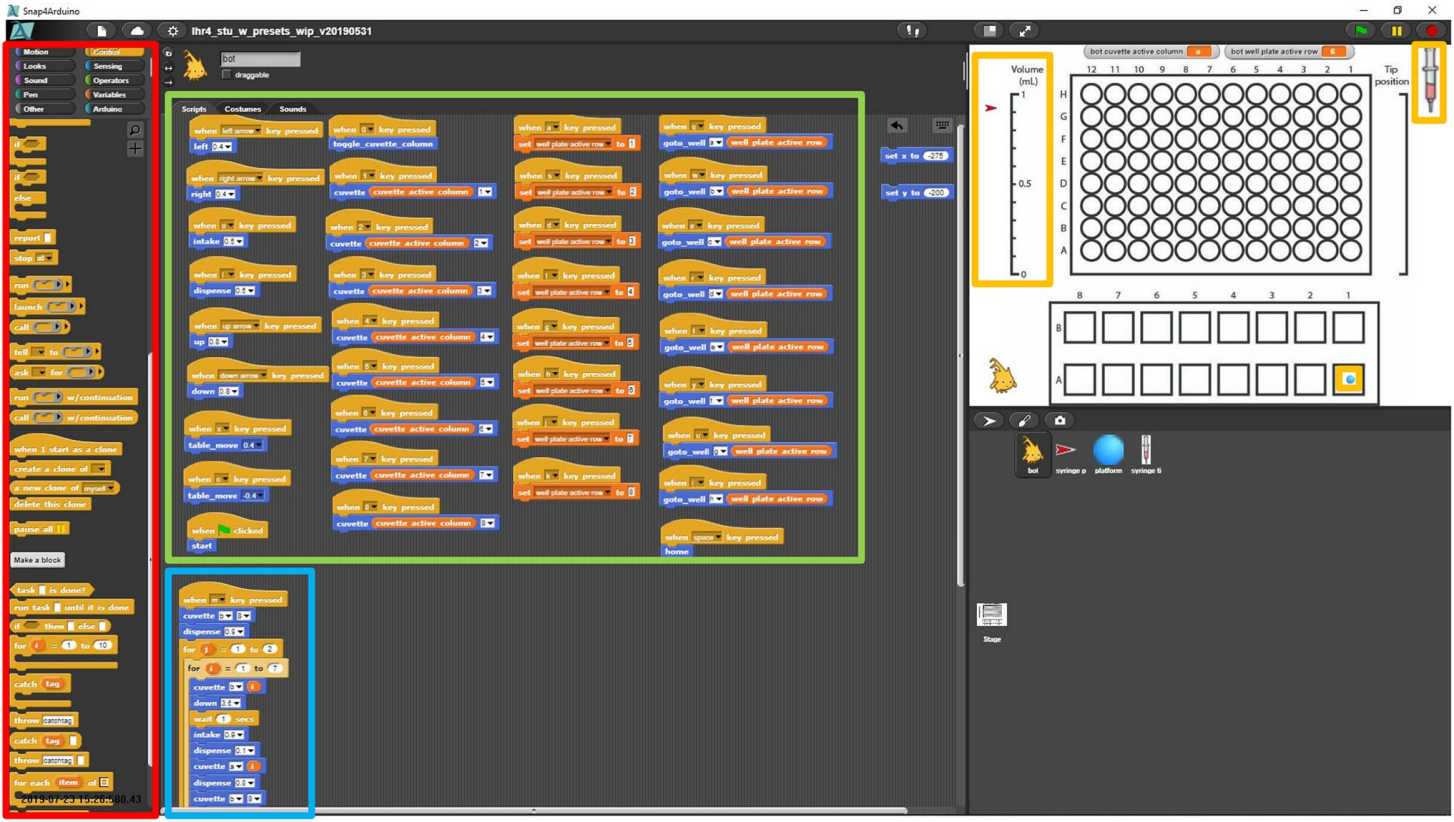
The programming interface. Red outline: Users can choose from basic programming constructs such as for-loops and move commands and drag them to the right to construct their own program. Green outline: The native set of commands for the LHR includes moving the pipette tip to specific locations on the stage, raising and lowering the pipette tip, and intaking and dispensing liquid. Blue outline: The beginning of the serial dilution command. Yellow outlines: Different markers indicate the location and state of the syringe. The red arrow on the left and the scale indicate how much liquid is contained in the syringe. The syringe image on the right indicates the current height of the syringe tip, and the blue dot indicates where on the stage the syringe is.

**Fig 5:**
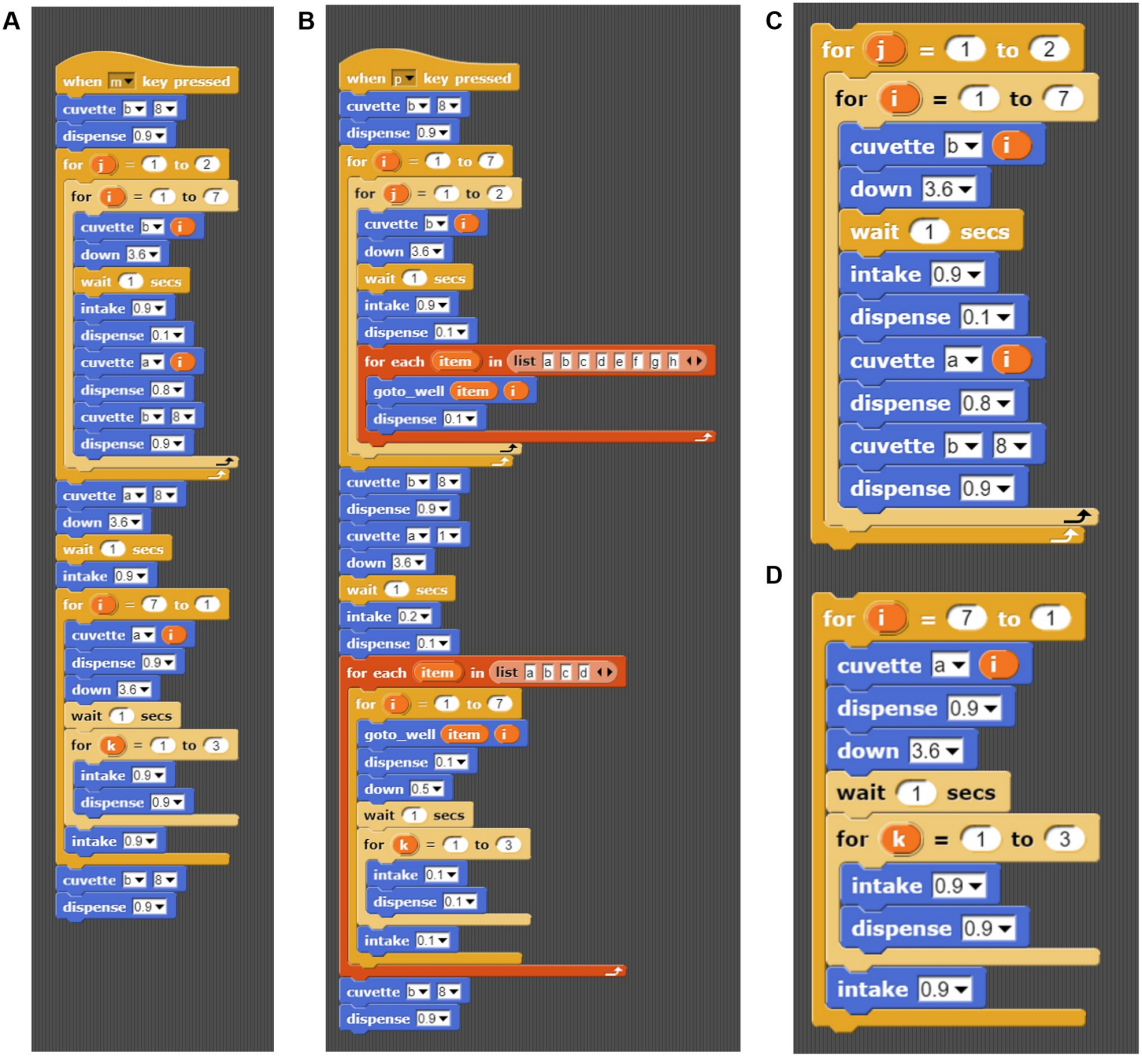
Examples of protocols featuring loops and iterative liquid handling operations. **A)** Serial dilution program for cuvettes. **B)** Serial dilution program for four rows of a 96-well plate. **C)** Zoom in of the first for-loop of the serial dilution program for cuvettes, which encapsulates the second for-loop. Note that the variables are marked in orange, the robot commands are marked in blue, and the for-loop visually encapsulates the entirety of its body. **D)** Third for-loop which encapsulates the fourth for-loop of the serial dilution program for cuvettes.

**Fig 6:**
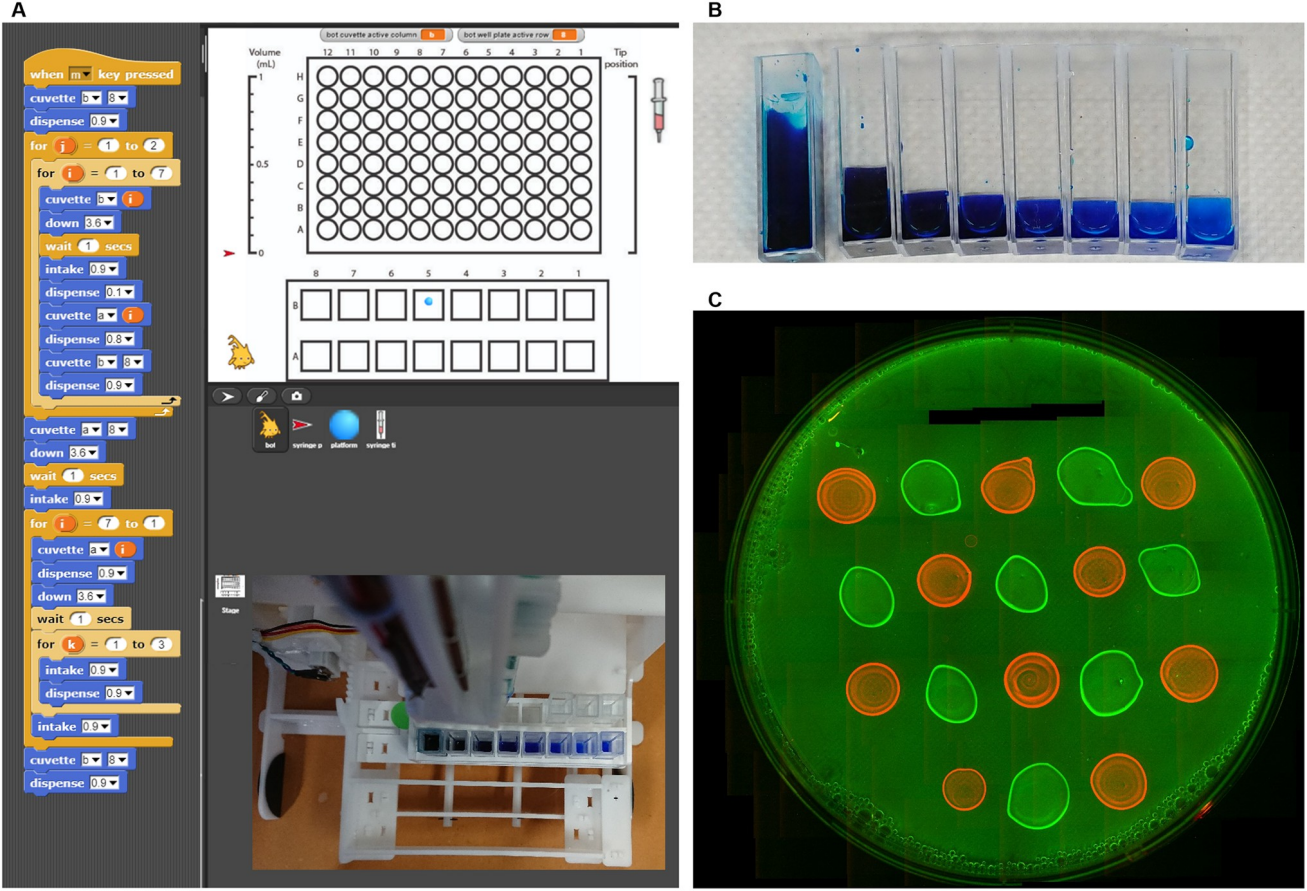
The robot was programmed to perform a serial dilution along a row of cuvettes, and to dispense 20 uL droplets of fluorescently-labeled E. coli onto an agar plate. **A)** The program for serial dilution was implemented through the Snap4Arduino interface. Inset is a top-down still image of the pipetting robot. **B)** The resulting cuvettes from the serial dilution. Each dilution decreased the dye concentration by about a factor of 3. The cuvette on the left was used to initialize the serial dilution. **C)** The fluorescent bacterial colonies after 1 hour of incubation.

For user interactions, we developed a traditional programming interface (a Python API) as well as a visual block-based programming interface (Figs 1B, 4, 5 and 6A) to enable programming by novices, similar to what was implemented in OpenLH [8]. The block-based interface was implemented in Snap4Arduino. Upon startup of the interface, a calibration routine is necessary to align the sensors. The base application includes commands for moving the stage forward and backward, moving the pipette tip left, right, up, and down, and intaking and dispensing liquid. These commands can be executed either by clicking on the program block with a mouse or by an associated key press. For example, pressing the left arrow will move the pipette tip left by an amount set by the user (the default is 0.4 cm). Users can also specify the distance of motion for each of the motion commands. The program also includes preprogrammed locations to which the pipette tip can be moved, corresponding with the initial layout of 16 cuvettes and a 96-well plate (Figs 1B, 4 and 6A).

In this interface, the tip location in the *x*-*y* plane is marked by a blue dot while the tip position in the *z*-axis is marked by the syringe picture to the right of the 96-well-plate diagram (Figs 1B, 4 and 6A). The amount of liquid contained within the syringe is marked by the pointer to the left of the 96-well plate diagram (Figs 1B, 4 and 6A, S1 and S3 Movies).

The Snap4Arduino interface allows users to drag and drop commands to create programs of their own (Figs 1B, 4 and 5, S3 Movie). Loops are indicated with visual brackets, which makes it easier for novice programmers to quickly understand their code. Variables and motion commands are represented in blocks of different colors.

### Applications: Liquid handling and biology experiments

Liquid intake and dispensing are handled by the 1 mL syringe, which can be purchased from Amazon (Plastic Syringe, Luer Slip, 1 mL, Amazon B00BQLJFYE). The syringe takes plastic pipette tips (Dispensing Needle, Plastic Tapered Pink 20 ga 0.024id x 1.25”, Amazon B001QQ9QH0). Both can be snapped into place and replaced as needed (Fig 2C). Our initial platform includes space for up to 16 standard cuvettes and standard 96-well plates (Fig 1A, Supplementary Information). For our experiments, we use Standard Polystyrene Cuvettes (3.5 mL, Amazon B00T5A64PQ) and Nest Scientific Polystyrene 96 Well Cell Culture Plate (Amazon B001QVE7C).

For the serial dilution demonstrations, food coloring was used (AmeriColor Beginner Soft Gel Paste Food Color 4 Pack Kit, Amazon B002L3RV9C) (Fig 6B, S1, S2 and S4 Movies). We also created agar plates and seeded fluorescent *E*. *coli* cell lines presenting mRuby and sfGFP (Null control strains from [22]) (Fig 6C).

### Demonstration 1: Serial dilution

We automated a routine for serial dilution in Snap4Arduino (Figs 5A and 6A, S1 Movie). The routine assumes that cuvette A8 is filled with the food coloring and the cuvettes in the second row (B1-B7) are filled with water. The routine dispenses an equal amount of water (1.6 mL) amongst the remainder of the cuvettes in row A. Then the pipette intakes 0.9 mL of the food coloring from cuvette A8 and dispenses it into A7. It mixes the liquid by passing it in and out of the syringe three times, then intakes 0.9 mL of the current cuvette, dispenses into the next cuvette in the row, and repeats this process for each subsequent cuvette. The result is a noticeable dye gradient (Fig 6B, S1 Movie).

This program demonstrates the possibility of using loops and establishing routines within the Snap4Arduino interface. The visual programming environment makes it relatively easy to understand the organization of the tasks and the loops (Figs 5 and 6A).

### Demonstration 2: Cell seeding on an agar plate

Fluorescent strains of *E*. *coli* (mRuby or sfGFP-tagged) were inoculated overnight for 10 hours. A fresh sterilized syringe and tip were used to withdraw 0.6 mL of the culture. The LHR then dispensed 0.02 mL droplets onto an agar plate in the configuration shown Fig 6D. To avoid contamination, a fresh pipette was used to dispense each strain of *E*. *coli*. To minimize pipette changes, all the mRuby-labeled *E*. *coli* droplets were dispensed before the sfGFP-labeled *E*. *coli* droplets.

### Teacher interest assessment, co-design, educational deployment with middle-school students

In order to assess interest, needs, and potential applications in educational settings, we invited teachers to visit our lab and provide feedback on the single-axis robot prototype (see S1 Movie). The purpose of this qualitative study was to provide feedback from relevant stakeholders and potential future users that could then be used to improve the final hardware and software design as detailed in this paper. Teachers were recruited by advertising via email to participants of a summer internship program for teachers at our institution. Anyone who was interested was then invited to meet. This study was performed according to Stanford IRB-18344.

We met with a total of 6 high school-level STEM teachers, all from the San Francisco Bay Area (specifically the Peninsula and the East Bay). One of the teachers taught biology and engineering; one taught only biology; two taught chemistry; one taught conceptual physics and computer science; and one taught computer science. Teachers met with us individually or in pairs. There were always at least two researchers present. Each meeting lasted approximately 1 hour. The meetings were structured as open interviews, where we demonstrated different functionalities of a single-axis prototype robot, and where we asked the teachers multiple questions regarding their teaching needs and what other ideas or comments they might have. We took notes on the teachers’ responses, either by typing directly into a computer or by writing on paper. Data was then qualitatively analyzed jointly by two researchers to identify points raised by multiple teachers, as well as to identify more unique perspectives. Overall, we believe that these teachers provided their honest opinion as we did not see any incentive for them to do otherwise, and we did not identify and conflicts of interest. We summarize the findings as follows:

Some examples of specific experiment activities and use cases that the teachers proposed include: titration experiments; growing organisms (e.g., algae, sea anemones); running experiments overnight or outside of classroom hours; quantifying the accuracy of pipette volume dispensing vs. humans with micropipettes (i.e., having students compare their pipetting technique to the robot); teaching students about measurements and tools in general; performing photosynthesis experiments comparing light/no-light conditions; color mixing; and testing for the presence of specific macromolecules (e.g., lipids). In general, activities proposed by the teachers tended to be ones that they already did in their classrooms, and they considered how a robot could make these experiments more feasible or more convenient to execute. Teachers with more curricular flexibility had greater interest in deploying the robot in their classes in comparison to teachers with less curricular flexibility.

Deploying the LHR into a classroom curriculum appealed to the teachers for the following reasons: (1) the ability to teach and foster programming at different levels based on student proficiency. The high-level interface and block-based programming environment (e.g., commands like “go to the well in row B of column 1 and lower the pipette by 0.25 cm”, or “intake 0.05 mL”) were appealing to teachers for whom programming was not the primary emphasis of the class, as it is simple enough for students without much programming experience. In contrast, the computer science teachers stated that their students could work with lower-level commands as well as the Python programming interface. (2) Engineering courses could have students design modifications to the robot, introducing fabrication technologies such as 3D printing, providing an entryway into the design process. Furthermore, teachers generally appreciated the “open” design of the robot, which keeps its structure and mechanisms exposed for students to see–thereby likely leading to a deeper understanding on how robots work. (3) Teachers saw significant potential for multidisciplinary teaching aligned with NGSS, as the robot could tie programming, engineering, and biology concepts together. It was specifically noted that for engineering students this type of LHR could serve as an introduction to life science applications.

In terms of limitations, the main concerns that teachers had were related to cost and robot precision: Even if students worked in teams with a robot, a single class would need about 10–15 LHRs, which likely represents a significant investment for schools. To justify this type of a purchase, the robots would have to be suitable for many different teachers and subject areas; and arranging courses to allow for that might take significant coordination. Teachers also commented on the limitations on positioning and volume dispensing accuracy, but they felt that such limitations could teach students about taking accuracy/reliability of tools in general into consideration; overall these limitations were not seen as a significant challenge for deployment.

Teachers had several suggestions for the robot design. While the biology/chemistry teachers wanted a ready-made robot citing a lack of time in the science class curricula for the LHR assembly process, the CS/engineering teachers believed that student assembly of the robot could be beneficial. Teachers also noted that for long-term or overnight experiments, it would be necessary to have the robot run on a Raspberry Pi (or similar) system, so that the robot could be run independently of the school computers, which may have time-specific shutdowns. Teachers also felt that ready-made lesson plans could make the LHR and other similar systems to be easier to adopt. These plans would ideally fit naturally into their curricula. All teachers wanted a robot with 3 positioning axes.

We also deployed the final LHR design inside of an educational setting and performed a use-case study with 6^th^ grade students; the results were published previously [21]. In summary, it was found that over a five-session curriculum these students were able to program the LHR to conduct a science experiment while also engaging into computational thinking (CT). Students identified increased precision in experimental procedures, time-efficiency, and easier debugging as the key advantages of this educational approach. This study provides a proof-of-concept curriculum on how such LHRs can bring CT and robotics to science classrooms, especially for chemistry and biology.

## Discussion

We presented a liquid handling robot that is open-source and DIY, similar to the EvoBot [15,16], featuring almost exclusively laser-cut or basic purchasable parts. Because the user has access to the blueprints of each individual laser cut piece, they have the flexibility to alter each module to suit their needs. The parts were designed to be snapped together; however, in designing and assembling this robot, we realized that variations in the laser cutters employed by different companies meant that each user would have to adjust the blueprints, specifically the tab-hole joints, on a case-by-case basis. Otherwise, the tabs might snap off during assembly, or not fit snugly enough. Joint stability was found to be greatly improved by gluing the joints after assembly. Assembly typically takes 1–3 days for the basic robot. This robot has relatively low price point: about $150 in parts–although additional costs occur if external vendors are used for tasks like laser cutting. Such external services can bring the cost to ~$400. We nevertheless estimate that a commercial LHR produced at large volumes could have a retail price on the order of ~$100. This makes it possible to employ this robot in non-professional settings, though it sacrifices speed and reliability compared to commercial high-end devices [9,17].

Table 1 provides a comparison of the DIY LHR with three other liquid handling robots that were designed with accessibility and affordability in mind but differed in intended application. There are clear trade-offs among the different designs. The DIY LHR stands out in its lower-cost fabrication, distributed manufacturing for the entire mechanical structure, and small footprint, while also maintaining acceptable performance for educational use. Other designs are easier to assemble and have greater accuracy. All robots are modularly expandable and modifiable to different extents, with the EvoBot likely providing the most customizability followed by the DIY LHR due to their hardware and software architectures.

**Table 1:** Comparison of four different LHRs that operate on standard plasticware (e.g., 96 well plates) and that were designed to be more accessible and lower cost than commercial research-grade products.

Robot name	DIY LH Robot	EvoBot	OpenLH	Lego LHR
Reference	This paper	[15,16]	[8]	[3]
Primarily intended application	Education (& research)	Life-science Research	HCI community; bio-enthusiasts	Education (& research)
Main hardware foundation	DIY electronics, laser-cut plastic parts	DIY electronics; metal and laser-cut plastic parts	Commercial robot, DIY pipettor	Lego EV3 kit
Accessible x-y range/ footprint	140x130 (mm x mm)*	600x400 (mm x mm)	250x250 (mm x mm)	260x180 (mm x mm)
x-y resolution	0.5 mm	0.1 mm	0.2 mm	2 mm
Volume resolution**	20 μl (0.2 μl)	10 μl (0.2 μl)	0.2 μl	2.5 μl (0.2 μl)
Software	Snap4Arduino code (or python)	Custom API (python)	Blockly code (or python)	Lego Mindstorms EV3 Software
Required technical assembly skills	Significant	Significant	Low-medium	Low
Production and assembly time	1–3 d	>1 d (?)	~ 1 h	~ 4 h
Price (parts + services)	$150 ($400***)	$600****	$1,000	$400

Some numbers are estimates as details were not directly stated or were not directly comparable. (*: The y-axis (sample stage) has a range of motion the could be arbitrarily extended. **:All systems could probably achieve volume resolutions down to 0.2 ul by just using a corresponding syringe, even though it was not tested for all robots. *** Depending on whether laser cut parts are made by a commercial vendor. **** System demonstrates multiple modular extensions that also affect the price.) HCI: Human-computer interaction.

The current DIY LHR design could be extended further and improved. For example, a camera could be added to enable more complex experiments and data collection, to aid pipette calibration, and to improve accuracy of pipette tip motion through visual servoing. Camera integration would also enable remote monitoring of the liquid handling robot which would then make it more suitable for applications in cloud laboratories [6,7]. This could be achieved with a Raspberry Pi (RPi) mini-computer and an attached camera module, as has been demonstrated in the past for other low-cost biology lab setups [6,7]. The minimal amount of liquid that can be reliably dispensed could be decreased to about 0.15 μL using a specialized syringe as we demonstrated previously [3]. Reproduction of the robot by others would be made easier by using standardized motor encoders in the LHR design. Overall, we found that the assembly of this LHR requires significant technical background knowledge and time, beyond that of the average K-12 science teacher. Potential future educational kits should be designed for easier assembly. Other processes could also be developed and programmed into the robot, such as a routine for switching pipette and/or cleaning syringe tips; effective cleaning and sterilization protocols for low-cost LHRs have been previously demonstrated [3,6,7].

We also highlight the use of a block-based programming user interface, which was intended to encourage novice programmers to develop their own protocols for repetitive tasks, and which was used in OpenLH [8]. The visual aspect of the programming language makes programs easier to build and read, clearly delimits the logical loops, and color-codes each block. This interface however was found to be limiting and/or cumbersome to more advanced programmers, which is why we also developed the Python API. Thus, the user does have access to a lower-level programming interface as well as higher-level user interfaces.

We already tested this LHR with children [21]. We expect that low-cost DIY LHRs would have many applications in different science and engineering classes and that it could be used to bridge many different subject areas, such as computer aided design, mechanical and electronic engineering, programming, and wet sciences (chemistry, biology), which would make such classes truly interdisciplinary. However, the specific needs of each class subject may vary. For example, for wet lab classes, robots should be optimized for ease of use, while student assembly and design modifications should be allowed for engineering classes. Low-cost, well-developed lesson plans for many different activities throughout the school year would make it much easier for teachers to use the LHR, though additional teacher training may still be required. More detailed studies with both teachers and students would further inform the development of educational applications for the LHR but are beyond the scope of this work (see [21] for the first user study with this LHR). Taken together, these design hardware and software choices could empower students to develop a ground-up understanding of how liquid handling robots work, and of robotics and automation in general.

In conclusion, LHRs and LHR kits could be produced at low cost, which would enable wide deployment in STEM educational settings, DIY spaces, and even professional lab science automation [3,6,7]. We expect that the presented design (or similar ones) will be most amenable to adapt as an educational tool, but specific research applications matching the systems performance and price point might exist as well. These technologies help students learn about automation, laboratory techniques, quantitative reasoning, mechatronics, systems engineering, and programming [3,8]. We suggest as next steps to (1) carry out additional targeted studies with middle and high school students and teachers on how to best integrate such technology into science or programming classes, and whether the inclusion of a robot into these courses changes student disposition towards STEM fields, and (2) to further develop this system towards a commercially available (educational) kit.

## Materials and methods

Materials and methods information are folded into the result section given that the paper is primarily concerned with device development and assessment.

User studies were approved by and performed according to Stanford IRB-18344. Participants gave informed written consent. No minors were included in this study.

We would like to thank R. Wright for help with manuscript preparation. We also thank L.W. for his help in the robot assembly trial.

## Supporting information

S1 Movie “UIandDilutionCombinedView”—A serial dilution performed on 8 standard 2.5 mL cuvettes demonstrating actuation of all four motors (three axis and syringe). Robot first fills standard 3.5 ml cuvettes with clear water and then performs a serial dilution experiment.
(MP4)

S2 Movie “SerialDilution-Top”—A serial dilution on a 96-well plate viewed from above. Because a standard 96-well plate is used, lower volumes are used in this dilution series (standard well volume 360 μL).
(MP4)

S3 Movie “ProgrammingDemo”—A basic demonstration of using the Snap4Arduino block-based programming interface. Demonstrates how block-based programming can be used to assemble loops.
(MP4)

S4 Movie “SingleAxisRobot”—A short demonstration of the initial single-axis robot. (MP4)
